# Clock Drawing Test – low accuracy in early hours

**DOI:** 10.1192/j.eurpsy.2022.1687

**Published:** 2022-09-01

**Authors:** K. Sejunaite, F. Gaucher, M. Riepe

**Affiliations:** Ulm University, Department Of Psychiatry And Psychotherapy Ii, Guenzburg, Germany

**Keywords:** clock drawing test, Cognitive disorders, early diagnostics

## Abstract

**Introduction:**

Early diagnostics of neurodegenerative disease and their comorbidities is linked to better treatment outcome and improved quality of life. The first patient assessment should lay strong foundations for the direction of the upcoming diagnostic procedure. Clock Drawing Test (CDT) is often used as an early screening instrument in geriatric patients presenting with cognitive disorders.

**Objectives:**

The goal of the present study was to evaluate diagnostic accuracy of the CDT in a geriatric cohort with mild cognitive difficulties.

**Methods:**

Out of a pool of in- and outpatient data presenting with subjective cognitive difficulties three diagnostic groups were formed – mild cognitive impairment, depressive disorder and healthy controls. CDT was scored using a quantitative scoring system with each aspect of the clock evaluated separately. CDT data was analysed for its discriminative value in early diagnostics of AD and DD.

**Results:**

Logistic regression produced a significant model with a low percentage of explained variance in both DD and AD groups. Same CDT items were significant predictors for DD and AD pathology. ROC curve inspection allowed only a poor discrimination capability for the significant predictors.

**Conclusions:**

Despite being a popular screening test, CDT is a poor choice for individuals presenting with a mild cognitive impairment. Using CDT alone might result in initial stages of neurodegeneration going undetected, thus depriving patients of early treatment options. Same error types were significant predictors in DD and AD. This indicates that CDT can detect a general impairment; however, an in-depth neuropsychological assessment is needed for differential diagnostics.

**Disclosure:**

No significant relationships.

